# The role of oxidative stress in 63 T-induced cytotoxicity against human lung cancer and normal lung fibroblast cell lines

**DOI:** 10.1007/s10637-018-0704-8

**Published:** 2018-11-29

**Authors:** Malgorzata Kucinska, Helena Mieszczak, Hanna Piotrowska-Kempisty, Mariusz Kaczmarek, Walter Granig, Marek Murias, Thomas Erker

**Affiliations:** 1 Department of Toxicology, Poznan University of Medical Sciences, Dojazd 30 Street, 60-631 Poznan, Poland; 2 Department of Immunology, Poznan University of Medical Sciences, Poznan, Poland; 3 Department of Medicinal Chemistry, University of Vienna, Vienna, Austria

**Keywords:** Reactive oxygen species, Lung cancer, Lung fibroblast, Antioxidative enzymes, Oxidative stress

## Abstract

It has been shown previously that molecules built on benzanilide and thiobenzanilide scaffolds possess differential biological properties including selective anticancer activity. In our previous study, we examined the cytotoxic activity and mechanism of action of the thiobenzanilide derivative N,N′-(1,2-phenylene)bis3,4,5–trifluorobenzothioamide (63 T) as a potential chemotherapeutic compound in an experimental model employing A549 lung adenocarcinoma cells and CCD39Lu non-tumorigenic lung fibroblasts. Since the results suggested oxidative stress as a co-existing mechanism of the cytotoxic effect exerted by 63 T on tested cells, studies involving the analysis of reactive oxygen species (ROS) generation and markers of oxidative stress in cells incubated with 63 T were carried out. It may be concluded that the selective activity of 63 T against cancer cells shown in our experiments is caused, at least in part, by the response of the tested cells to 63 T mediated oxidative stress in both tested cell lines.

**Electronic supplementary material:**

The online version of this article (10.1007/s10637-018-0704-8) contains supplementary material, which is available to authorized users.

## Introduction

Oxidative stress is a fundamental concept in redox biology and medicine. It is defined as “an imbalance between oxidants and antioxidants in favor of the oxidants, leading to a disruption of redox signaling and control and/or molecular damage” [1]. Over the past several years, it has been shown that oxidative stress could be related to the pathomechanism of neurodegenerative [2], cardiovascular [3], and metabolic disorders [4] as well malignant diseases [5]. Numerous studies shed light on the important role of the overproduction of reactive oxygen species (ROS) in the development of different types of cancer, as this increases DNA mutations and damage, increasing the tendency for alterations in the genome and cancer cell growth and preventing cell death [6, 7]. Moreover, it is well known that a moderate increase of ROS production may stimulate cell proliferation while an excessively high increase corresponds to several abnormalities and decreases viability [8]. Although an antioxidant treatment is considered as chemoprevention which may potentially protect from oxidative injuries, it should be emphasized that, depending on several circumstances, the use of antioxidants may have an adverse effect by promoting cancer cell survival [9, 10].

In general, ROS levels are higher in cancer cells compared to non-transformed cells [10], thus targeting cancer cells by boosting ROS generation may enhance the cytotoxic effects of anticancer agents. This phenomenon has important implications for pro-oxidant therapies, including radiotherapy, photodynamic therapy and chemotherapeutic agents which are based on oxidative stress induction [8, 11]. However, cancer cells adapt to continuous oxidative stress through several mechanisms such as the upregulation of antioxidative enzymes, intracellular antioxidants and reprogramming metabolic pathways [12, 13]. Indeed, several studies have shown that a high level of intracellular antioxidants and antioxidative enzymes could be connected with the resistance of cancer cells to ROS-inducing agents [14–16]. The main ROS-detoxifying enzymes including superoxide dismutase (SOD), catalase (CAT) and glutathione peroxidases (GPx) play a role in the breakdown of the superoxide radical (O_2_^-.^), hydrogen peroxide (H_2_O_2_) as well other hydroperoxides [17]. Other proteins like peroxiredoxins, thioredoxins (Trx), glutaredoxins (Grx), as well low-molecular-weight antioxidants like glutathione (GSH), tocopherol and ascorbate, are involved in ROS scavenging and, thus, prevent oxidative modification of biologically important molecules [17]. Beyond ROS, an antioxidant defense is also important for maintaining the levels of reactive nitrogen species (RNS), a family of highly reactive, nitrogen-bearing molecules including the nitric oxide radical (NO or NO˙), nitrogen dioxide radical (NO_2_˙), nitrite (NO_2_^−^) and peroxynitrite (ONOO^−^) [18]. Considering that ROS production is lower in normal cells and the basal level of antioxidative enzymes is different, a therapeutic approach that targets the antioxidant system might significantly increase selectivity and enhance ROS-mediated cancer cell death [12].

**Fig. 1:**
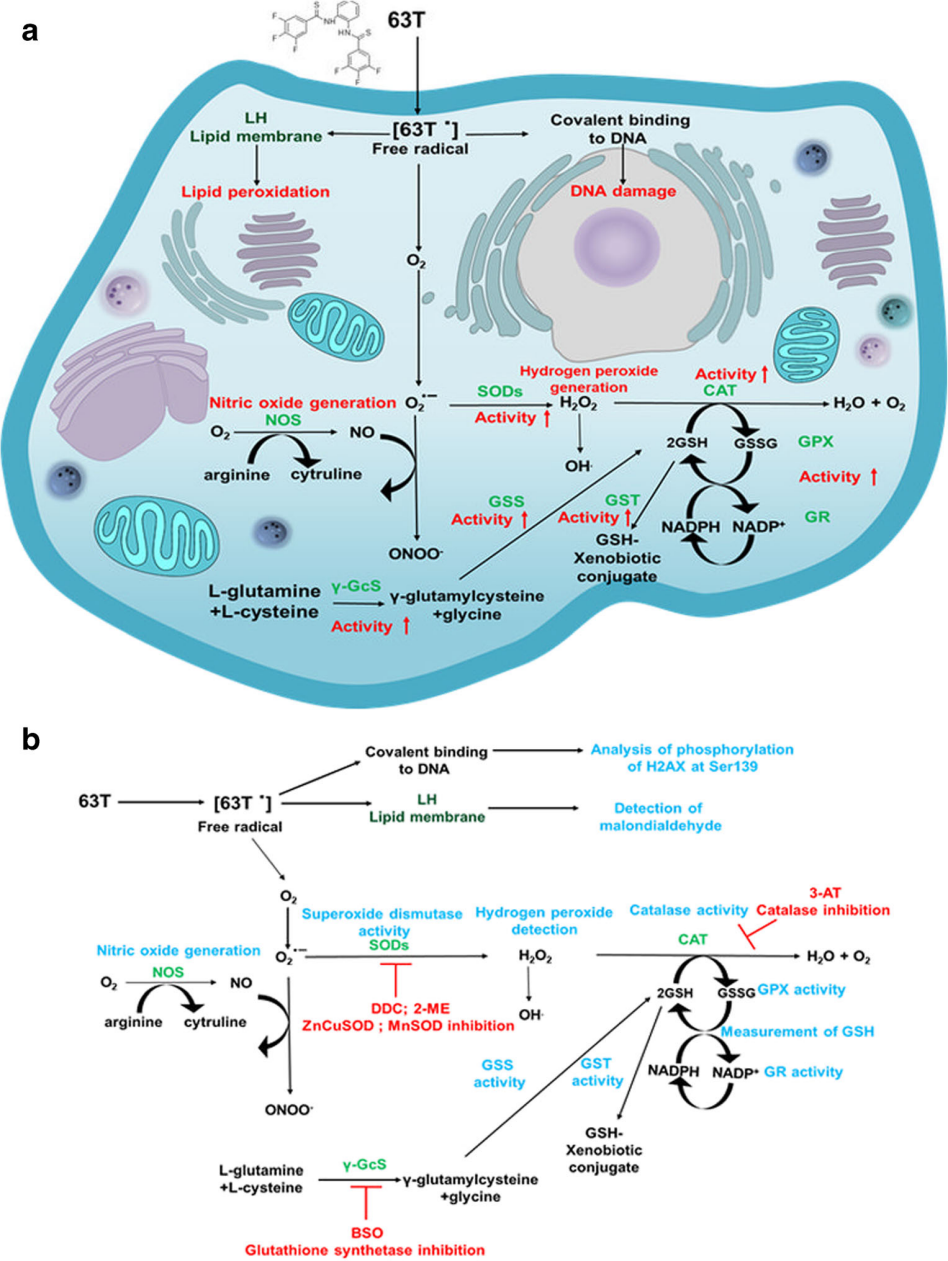
**The proposed mechanism of anticancer activity mechanism of 63 T as an oxidative stress-induced compound (a). The schematic overview of performed experiments which were designed to test the hypothesis that 63 T may act as a pro-oxidative compound (b).** Abbreviations: γ-GcS, glutathione synthetase; 2-ME, 2-methoxyestradiol; 3-AT, 3-Amino-1,2,4-triazole; BSO, L-buthionine-sulfoximine; CAT, catalase; DDC, diethyldithiocarbamate; GPx, glutathione peroxidase; GR, glutathione reductase; GSH, glutathione; GSS, Glutathione synthetase; GSSG, glutathione disulfide; GST, glutathione-S-transferase; H_2_O_2_, hydrogen peroxide; NO, nitric oxide; NOS, nitric oxide synthetase; O2^.-^, superoxide; OH^.^, hydroxyl radical; ONOO-, peroxynitrite; ROS, reactive oxygen species; SOD, superoxide dismutase

To date, molecules built on benzanilide and thiobenzanilide scaffolds have been found to exert promising spasmolytic [19], antibacterial [20], antimycotic [21], antifungal [22] and estrogenic [23], as well anticancer [24, 25], activity. In previous studies, the cytotoxic activity of the thiobenzanilide derivative N,N′-(1,2-phenylene)bis3,4,5–trifluorobenzothioamide (63 T) has been broadly reviewed for a variety of cancer cell lines including breast and lung cancer [23]. Particularly noteworthy is that 63 T exhibited significant anticancer activity against the lung cancer cell line A549 with a half maximal inhibitory concentration (IC_50_) of >1 μM, 0.41 μM and 0.36 μM after 24 h, 48 h and 72 h, respectively [25]. Moreover, experiments using the normal human fibroblast (CCD39Lu) and cancer cell lines confirmed the selective cytotoxic effect of 63 T. The calculated selectivity index after 48 h was 2.7 (IC_50_CCD39Lu/IC_50_A549), which is superior to results obtained in similar experiments for clinically approved drugs [25]. Our previous data showed that 63 T selectively induces cancer cell death in the caspase independent pathway, and the autophagic dose-dependent processes may be involved in the mechanism of cell death [25]. However, various aspects related to molecular targets and the antitumor effects of 63 T are still unknown [25]. In line with our preliminary studies, which indicated the generation of oxidative stress as a possible antitumor mechanism of 63 T, further experiments were designed to explore the anticancer effect of the aforementioned benzanilide derivative as an oxidative stress-inducing agent. In the current work, ROS generation, lipid peroxidation, detection of H_2_O_2_ and NO, the activity of antioxidative enzymes (mitochondrial superoxide dismutase [MnSOD], CAT, glutathione-S-transferase [GST], GPx) as well the status of glutathione in cancer and normal cells have been assessed. Additionally, we investigated the effects of antioxidative enzyme inhibitors on 63 T cytotoxic activity and DNA damage as a potential effect of oxidative stress. A general overview of the performed experiments and the aims of this study are presented in Fig. 1.

## Materials and methods

### Chemicals

All chemicals used in experiments were obtained from Sigma-Aldrich (St. Louis, Mo, USA) unless otherwise stated.

### Cell culture

All experiments were performed using two cell lines: A549 (human lung adenocarcinoma) and CCD39Lu (normal human lung fibroblast). Both cell lines were obtained from the European Collection of Authenticated Cells Cultures (ECACC, Salisbury, UK) and cultured in Dulbecco’s Modified Eagle’s Medium (DMEM) without phenol red supplemented with 10% FBS, 1% penicillin/streptomycin and 1% L-glutamine at 37 °C Gibco Invitrogen Corp. (Grand Island, NY, USA), in a humidified atmosphere containing 5% CO_2_.

### Tested compound

**Fig. 2:**
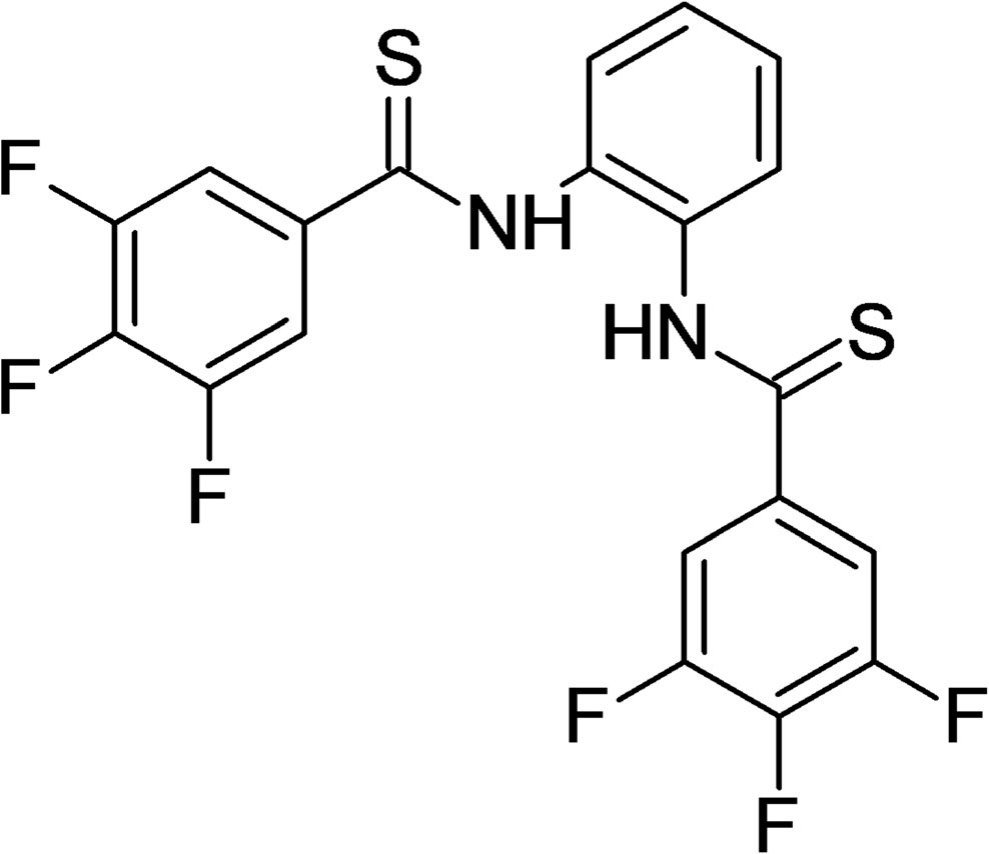
**Chemical structure of tested compound** 3,4,5-trifluoro-N-[2-[(3,4,5-trifluorobenzenecarbothioyl) amino]phenyl]benzenecarbothioamide – 63 T

The tested compound, 3,4,5-trifluoro-N-[2-[(3,4,5-trifluorobenzenecarbothioyl) amino]phenyl]benzenecarbothioamide (63 T) (PubChem CID: 51346886) (Fig. 2), was synthesized as described previously [19]. The compound was selected from the group of benzanilides as was previously described by our group. The stock solution of the tested compound 63 T (1 mM) was prepared by dissolving it in DMSO and then storing it in the dark at -20 °C.

The doses of 63 T and incubation timepoints were selected based on a viability study [25]. In previous research, we determined the IC_50_ values of 63 T for each cell line incubated with the tested compound at concentrations ranging from 0.03 μM to 1 μM for 24 h, 48 h, and 72 h of incubation. The IC_50_ for A549 was 0.41 μM and 0.36 μM for 24 h, 48 h and 72 h of incubation respectively, while CCD39Lu cells were less sensitive and the IC_50_ was 0.7 μM for 72 h of incubation [25]. Therefore, to better describe the activity of 63 T, we decided to use three different doses referred to as high cytotoxic (1 μM), moderate (0.5 μM) and mild (0.25 μM) and three incubation time points.

### Detection of ROS

Two different methods were used to investigate the influence of 63 T on cellular radical generation: fluorescence microscopy and flow cytometry. For this purpose, the non-fluorescent 2′,7′-dichlorodihydrofluorescein diacetate (DCFH-DA) was employed. DCFH-DA after de-esterification to 2′,7′-dichlorodihydrofluorescein (DCFH), is further oxidized to fluorescent 2′,7′-dichlorofluorescein (DCF) by ROS as well RNS. For microscopic analysis, 25 × 10^4^ cells/well were seeded into each of the wells of a 12-well plate. After 6 h and 24 h incubation with 63 T at concentrations of 0.25 μM, 0.5 μM and 1 μM, the cells were washed with phosphate buffered saline (PBS) and DCFH-DA (10 μM in PBS) was added to each well. The plates were incubated for 30 min at 37 °C. Then, the plates were washed twice with PBS and observed under a fluorescence microscope (Nikon Eclipse TS100 microscope with attached fluorescence unit model C-SHG and digital camera DS-SMc).

For flow cytometry analysis, cells were seeded in a 25 cm^2^ flask and incubated overnight under cell culture conditions. Then 63 T was added at concentrations of 0.25 μM, 0.5 μM and 1 μM and incubated for 24 h. DMSO was used as the negative control and the concentration did not exceed 0.1%. For the positive control, 1 μM of cumene hydroperoxide (CHPO) was used. Cells were harvested by trypsinization, then suspended in a DCFH-DA solution in PBS (0.1 μM) and incubated for 30 min at 37 °C in dark conditions. Cells were centrifuged at 1300 rpm for 5 min and washed twice with PBS. ROS generation was determined by FACS Calibur (Becton & Dickinson, USA). The results are presented as the mean ± SD from three independent experiments.

### Lipid peroxidation measurement

Lipid peroxidation was measured using the Lipid Peroxidation (MDA) Assay Kit (Sigma-Aldrich, St. Louis, MO, USA) according to the manufacturer’s instructions. Extensive oxidative species generation can lead to the formation of malondialdehyde (MDA) as a result of the peroxidation of polyunsaturated fatty acids (PUFAs) which contain at least three double bonds. MDA can react with thiobarbituric acid to form a colorimetric, as well a fluorescent, product which is a well-known biomarker of the peroxidation of polyunsaturated lipids [26]. The A549 and CCD39Lu cells were seeded at a density of 5 × 10^5^ cells per well. The cells were treated with 63 T at concentrations of 0.25 μM, 0.5 μM and 1 μM for 24 h. Then, cells were lysed on ice using MDA lysis buffer containing 3 μL of butylated hydroxytoluene (BHT) and samples were centrifuged at 13,000×g for 10 min. Next, a thiobarbituric acid (TBA) solution was added and incubated at 95 °C for 60 min. The samples were chilled to room temperature in an ice bath for 10 min and transferred into a black bottom 96-well plate. The fluorescence of MDA-TBA adducts was measured using microplate reader Tecan Infinity 200 (Männedorf, Switzerland) at 532 nm and 553 nm for excitation and emission, respectively. The MDA solution was used as a standard at concentrations of 0.4 nM, 0.8 nM, 1.2 nM, 1.6 nM and 2.0 nM.

### Detection of DNA damage

Double stranded breaks (DSBs) can be associated with the phosphorylation of histone H2A variant H2AX. Because phosphorylation of H2AX at Ser^139^ (γH2AX) is abundant, fast, and correlates well with each DSB, it is one of the most sensitive markers used to determine DNA damage [27].

DNA damage after 63 T treatment was detected using an EpiQuik™ In Situ DNA Damage Assay Kit (Epigentek, Farmingdale, NY, USA) according to the manufacturer’s protocol. A549 and CCD39Lu cells were seeded on 96-well plates at a density of 2 × 10^4^ cells per well and incubated overnight. Then, cells were exposed to 63 T at concentrations of 0.25 μM, 0.5 μM and 1 μM for 24 h. Cells were fixed using paraformaldehyde, permeabilized and blocked. The phosphorylation of H2AX at serine^139^ was detected by the primary anti-phospho H2AX^Ser139^ antibody and HRP conjugated secondary antibody. The absorbance was measured at 490 nm using a plate reader (Biotek Instruments, Elx-800). The results are presented as a percent of control (mean ± SD from two independent experiments).

### Evaluation of H_2_O_2_ production

H_2_O_2_ was detected using a Hydrogen Peroxide Cell-based Assay Kit according to the manufacturer’s protocol. A549 and CCD39Lu cells were seeded at a density of 2 × 10^4^ cells per well on 96-well plates and incubated overnight. Next, the tested compound 63 T was added at concentrations of 0.25 μM, 0.5 μM and 1 μM for 12 h and 24 h under cell culture conditions. Subsequently, plates were centrifuged at 400 x g for 5 min, and 80 μl of the medium was transferred to another plate and the assay buffer and reaction mixture were added to the samples and standards. Plates were incubated with gentle shaking on an orbital shaker for 15–30 min at room temperature. The resorufin fluorescence produced was measured using a Tecan Infinite 200Pro (excitation: 530 nm; emission: 590 nm). The protein concentration was measured using the BCA Protein Assay.

### Measurement of CAT activity

CAT activity was measured using a commercially available kit purchased from Cayman Chemicals (Ann Arbor, MI, USA). The method was based on the reaction of CAT with methanol in the presence of an optimal concentration of H_2_O_2_. The formaldehyde produced in this reaction was measured spectrophotometrically at 540 nm with purpald (4-amino-3-hydrazino-5-mercapto-1,2,4-triazole) as the chromogen. Purpald specifically forms a bicyclic heterocycle with aldehydes, which upon oxidation changes from colorless to a purple color. A549 and CCD39Lu cells were seeded in 25 cm^2^ flasks and incubated under cell culture conditions. Then 63 T was used at concentrations of 0.25 μM, 0.5 μM and 1 μM and incubated for 12 h, 24 h, and 48 h. Cells were collected using a rubber policeman and centrifuged (1300 rpm, 10 min at 4 °C). Cells were sonicated in 1 ml of cold buffer and centrifuged at 10,000 rpm for 15 min at 4 °C and supernatants were transferred into the new tubes. As a standard, formaldehyde was used. The samples and standards were mixed with methanol and an assay buffer, and then H_2_O_2_ was added and incubated on a shaker for 20 min at room temperature. To terminate the reaction, potassium hydroxide was added, and then CAT purpald (chromogen) was added to each well and plates were incubated for 10 min on a shaker. Next, potassium periodate was added and incubated for 5 min on a shaker. The absorbance at 540 nm was measured using a BioTek plate reader.

### Assessment of MnSOD activity

MnSOD activity was analyzed using a Manganese Superoxide Dismutase Assay HCS232 (Millipore) according to the manufacturer’s protocol. A549 and CCD39Lu cells were seeded at a density of 2 × 10^4^ cells on 96-well plates and incubated under cell culture conditions. On the next day, the 63 T was added at concentrations of 0.25 μM, 0.5 μM and 1 μM and incubated for 24 h and 48 h. As a positive control, etoposide at a concentration of 100 μM was used. After incubation, cells were fixed at room temperature for 30 min, washed and the primary antibody was added for 1 h at room temperature. Then, cells were washed three times and incubated for 1 h with the secondary antibody/Hoechst dye. Cells were washed four times and analyzed under a fluorescence microscope (Nikon Eclipse TS100 microscope with attached fluorescence unit model C-SHG and digital camera DS-SMc).

### Measurement of GSH content

The GSH level in the A549 and CCD39Lu cells incubated with 63 T was measured using ThioGlo-1 (Calbiochem, San Diego, CA). ThioGlo-1 is a maleimide reagent, which produces highly fluorescent adducts upon reaction with thiol groups, chiefly with intracellular GSH [28]. Briefly, A549 and CCD39Lu cells were seeded in 96-well plates at a density of 2 × 10^4^ cells per well and incubated overnight. Cells were treated with 63 T alone or with 63 T and buthionine sulphoximine (BSO) - a gamma-glutamylcysteine synthetase (γ-GCS), GSH synthesis inhibitor (1.125 mM) for 24 h and 48 h under cell culture conditions. Then, cells were lysed and incubated for 30 min at 4 °C. 50 μL of lysates were transferred to white 96-well plates, and 50 μL of the freshly diluted ThioGlo-1 (Calbiochem, San Diego, CA) reagent in PBS (5 μM) was added to the standards and samples. Plates were mixed using a plate shaker and incubated in the dark for 5 min and measured (λ_EX_ 384 nm, λ_EM_ 513 nm) using a microplate reader, Tecan Infinity 200 (Männedorf, Switzerland). The total GSH was calculated using a standard curve with GSH standard solution of concentrations (0.3–1 μM). The data are expressed as the amount of GSH per total protein (mean ± SD) from two independent experiments.

### Evaluation of GST and GPx activity

After exposure, the cells were scraped, washed twice with ice-cold PBS and centrifuged at 200 x g for 5 min at 4 °C. Then the cells were resuspended in PBS with 0.05% Triton X-100 (*v*/v) and sonicated three times for 5 s intervals in ice-cold ultrapure water. After that, the suspension was centrifuged at 10,000 x g at 4 °C, and the supernatant aliquots were stored at −80 °C. GST activity measurement was based on the spectrophotometric determination of 1-chloro-2,4-dinitrobenzene (CDNB) conjugate formed in a GSH coupled reaction at 340 nm. The activity was expressed as nmol CDNB conjugated with GSH per min per mg protein and compared with activity measured in the control cells. GPx activity was determined by measuring the disappearance of β-Nicotinamide adenine dinucleotide 2′-phosphate reduced tetrasodium salt hydrate (NADPH) at 340 nm, as the substrate cumene peroxide was used. Activity was calculated and expressed as nmol of NADPH oxidized per min per mg protein [29].

### NO production

NO production was determined using cell-permeable 4-Amino-5-methylamino-2′,7′-difluorofluorescein diacetate (DAF-FM DA). In living cells, DAF-FM DA is rapidly transformed into water-soluble 4-Amino-5-methylamino-2′,7′-difluorofluorescein (DAF-FM) by cytosolic esterases.

A549 and CCD39Lu cells were seeded at a density of 2 × 10^4^ cells per well on white 96-well plates and incubated overnight. 63 T was added at concentrations of 0.25 μM, 0.5 μM and 1 μM for 12 h and 24 h under cell culture conditions. Next, cells were washed twice with PBS, and DAF-FM DA in a serum-free medium (final concentration 10 μM) was added to each well. Cells were incubated for 30 min under cell culture conditions. Then cells were washed twice with PBS and incubated for another 15 min. The fluorescence of the samples was measured using a Tecan Infinite 200Pro (excitation: 485 nm; emission: 535 nm). The protein concentration was measured using the BCA Protein Assay.

### Treatment of inhibitors – Impact on antioxidative enzymes

A549 and CCD39Lu cells were seeded at a density of 2 × 10^4^ cells per well in 96-well plates and incubated overnight under cell culture conditions. Cells were treated with 63 T at concentrations of 0.25 μM, 0.5 μM and 1 μM and with antioxidative enzymes: 2-methoxyestradiol (2-ME) – a MnSOD inhibitor – at a concentration of 1 μM, diethyldithiocarbamate (DDC) – a CuZn-superoxide dismutase (CuZnSOD) inhibitor – at a concentration of 10 μM, 3-Amino-1,2,4-triazole (3AT) – a CAT inhibitor – at a concentration of 5 mM, and BSO – a γ-GCS (GSH synthesis) inhibitor – at a concentration of 1.5 μM. Inhibitors and their concentrations were selected after literature search [30] and after preliminary studies. Cells were incubated for 24 h and 48 h. DMSO was used as the negative control. The cell viability was measured using the MTT assay.

### Protein measurement

Protein concentration was measured according to the Lowry method using a Bio-Rad Protein Assay Kit (Bio-Rad Laboratories, Melville, NY, USA). Bovine serum albumin (BSA) was used as the standard.

### Statistical analysis

Statistical analyses were carried out using one-way ANOVA with Dunnett’s multiple comparison tests. The results are presented as the mean ± SD from two or three independent experiments. The values were calculated using GraphPad Prism version 5.00 for Windows (GraphPad Software, San Diego, CA, USA).

## Results

### Detection of ROS

**Fig. 3:**
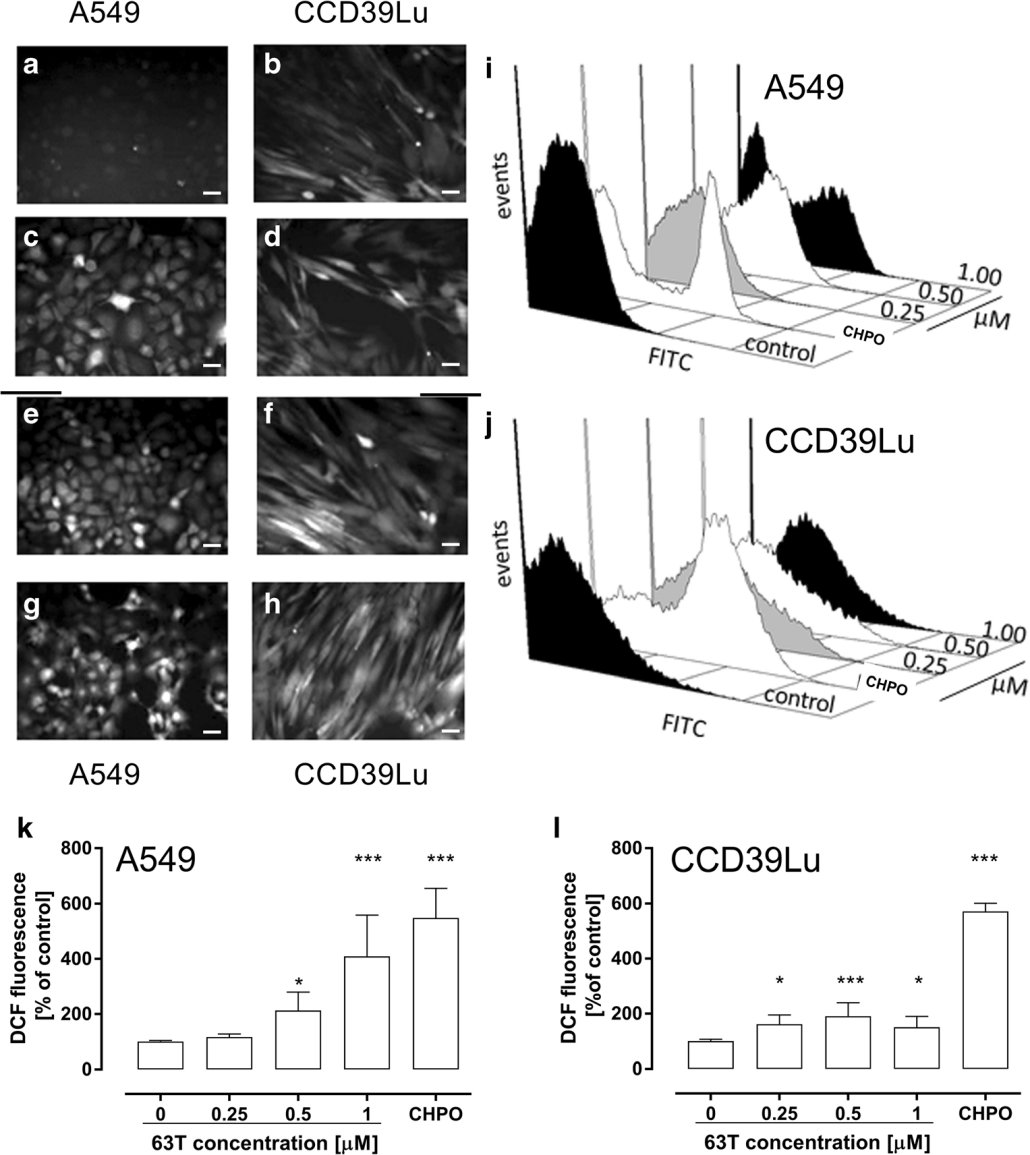
**Induction of oxidative stress in A549 and CCD39Lu cells incubated with 63 T.** The oxidative stress generation was analyzed by FACS and fluorescence microscopy after incubation with 63 T for 24 h, followed by H_2_DCF-DA staining. As a positive control, cumene hydroperoxide was used. The representative photographs are presented in the panel on the left of the figure: A549 cells: **a** – control, **c** – 0.25 μM, **e** – 0.5 μM, **g** – 1 μM; CCD39Lu cells, **b** – control, **d** – 0.25 μM, **f** – 0.5 μM, **h** – 1 μM. Scale bar corresponds to 100 μM. The representative flow cytometry histograms and corresponding charts are presented for A549 **(i, k)** and CCD39Lu **(j, l)**, respectively. Statistical significance is indicated with asterisks: *p < 0.05, **p < 0.01, ***p < 0.001

A DCFH-DA assay was employed to investigate the influence of 63 T on cellular radical generation. ROS generation was evaluated using fluorescence microscopy and flow cytometry. The flow cytometry showed a significant dose-dependent increase of the ROS level in A549 cells. Compared with the control, 0.5 μM and 1 μM of 63 T increased the ROS generation by 113% and 309%, respectively. Although the increase of ROS in CCD39Lu cells was also observed for doses of 0.25 μM, 0.5 μM and 1 μM by 61%, 90%, 50% respectively, compared with the control, the ROS generation was lower compared to A549 cells. Increased ROS production was also observed by fluorescence microscopy and, interestingly, “hot spots” were observed in these pictures (Fig. 3). These results suggest that the cytotoxic effect of 63 T is dependent on the generation of ROS. Furthemore, our results demonstrated that 63 T may target preferentially cancer cells by ROS-mediated mechanism.

### Lipid peroxidation measurement

**Fig. 4:**
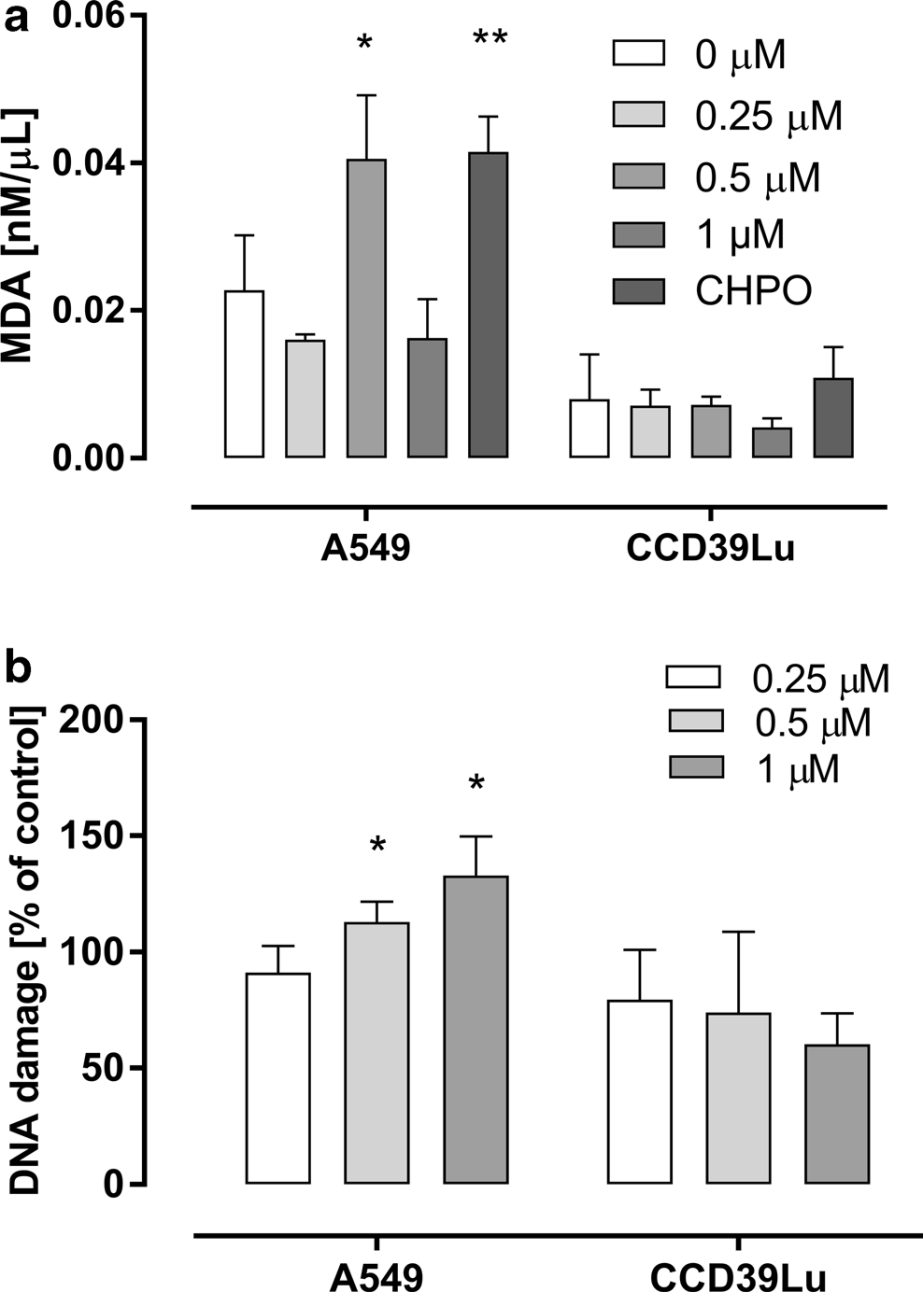
**Lipid peroxidation and DNA damage in cells treated with 63 T.** Malondialdehyde formation as a marker of lipid peroxidation in cells incubated with 63 T is presented in **panel a** while phosphorylation of H2AX at Ser^139^ as a marker of DNA damage in cells incubated with 63 T is shown in **panel b**. Both analysis were performed after 24 h of incubation with 63 T. Statistical significance is indicated with asterisks: *p < 0.05

ROS may easily react with lipids and, thus, lipid peroxidation is one of the most widely used markers of free radical formation. The peroxidation of membrane lipids may dramatically change the physical properties of lipid bilayers such as lipid-lipid interactions, ion gradients, membrane fluidity, and permeability [31]. In our study, MDA, the well-known biomarker of lipid oxidation, was measured after the cells were incubated with 63 T. At a concentration of 0.5 μM, the MDA level in A549 cells was significantly increased and was similar to the level measured in cells incubated with CHPO (the positive control). In contrast, 63 T at a concentration of 1 μM induced lipid peroxidation comparable to that observed for the control cells (Fig. 4a). Interestingly, no significant difference in MDA level was observed for the CCD39Lu cell line. These findings suggest that cell lipid peroxidation may be selectively induced in cancer cells which are more sensitive to oxidative stress induced by 63 T.

### Evaluation of DNA damage

Oxidative stress-induced DNA damage is well known as a major cause leading to cell death. We found that incubation with 63 T led to DNA damage in A549 cells at concentrations of 0.5 μM and 1 μM (Fig. 4b). By contrast, in CCD39Lu cells, DNA damage did not occur **(**Fig. 4b**).** These results demonstrate that 63 T may induce DNA damage at the mild and high doses within 24 h of treatment.

### H_2_O_2_ measurement

**Fig. 5:**
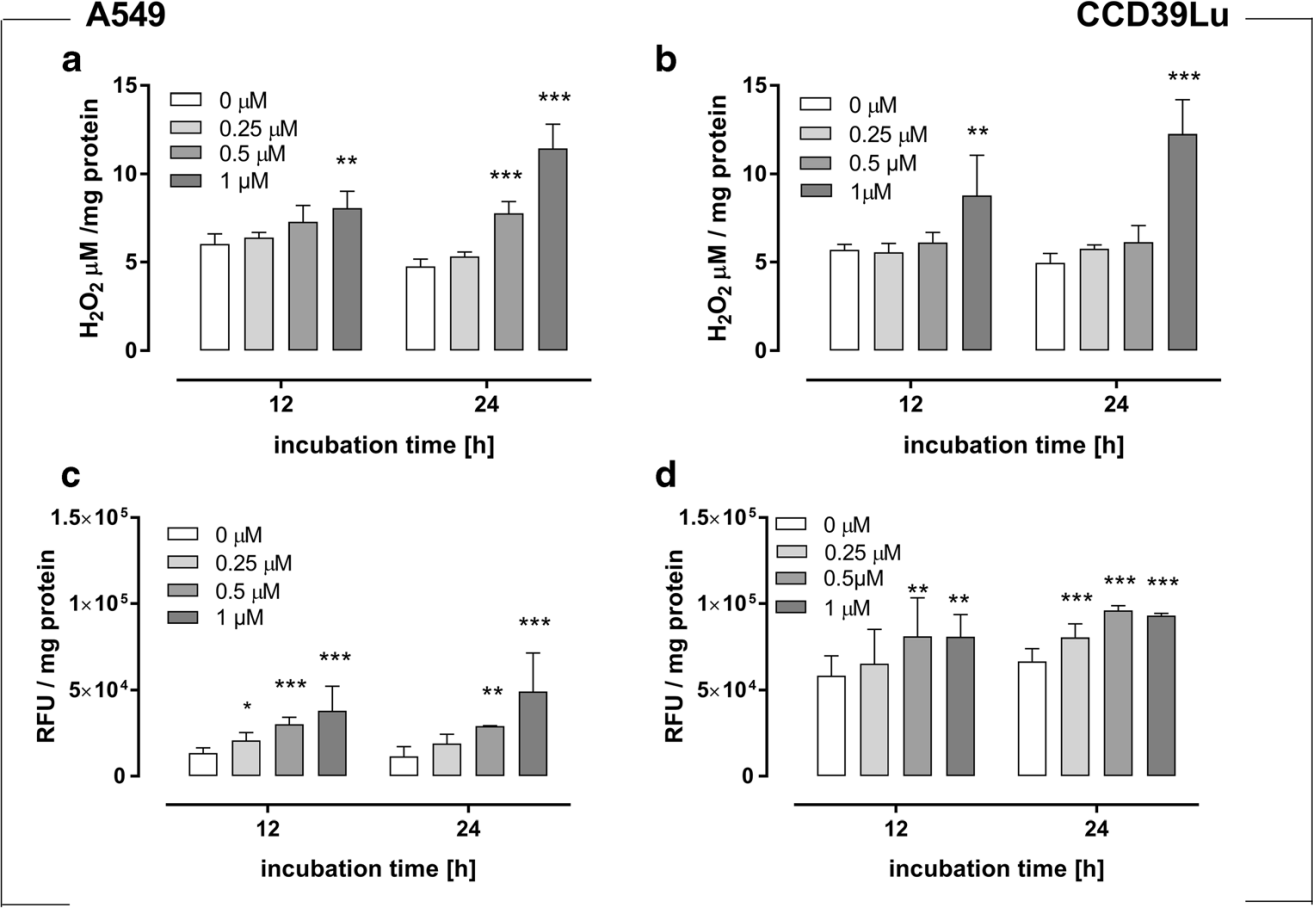
**NO and H**_**2**_**O**_**2**_**generation in A549 and CCD39Lu after incubation with 63 T.** Hydrogen peroxide generation after 12 h and 24 h incubation with 63 T is shown for A549 (**a**) and CCD39Lu (**b**) cells. Results are presented as μM/mg protein. The nitric oxygen production was measured with DAF after 12 h and 24 h incubation with 63 T in A549 (**c**) and CCD39Lu (**d**) cell lines. For both analyses, data are presented as mean ± SD from two separated experiments. Statistical significance is indicated with asterisks: *p < 0.05, **p < 0.01, ***p < 0.001

H_2_O_2_ is one of the major redox metabolites involved in physiological and pathological processes such as redox sensing, signaling and redox regulation. Our previous study has shown that treatment with 63 T increased the caspase protein level. Therefore, we tested whether H_2_O_2,_ as a main substrate for CAT, plays an important role in 63 T-mediated oxidative stress induction. It was found that incubation with 63 T at a concentration of 1 μM for 12 h increased the level of H_2_O_2_ (by 33% of control). The level of H_2_O_2_ measured after 24 h was significantly increased in cells incubated with 63 T at a concentration of 0.5 μM (by 63% of control) and 1 μM (by 140% of control). In CCD39Lu fibroblasts, a significantly higher level of H_2_O_2_ was present after 12 h and 24 h only in cells incubated with 63 T at a concentration of 1 μM (by 54% and 146% of control, respectively) (Fig. 5a and b).

### Nitric oxide production

For both tested cell lines, incubation with 63 T at a concentration of 0.5 μM and 1 μM led to an increase in the level of NO (Fig. 5c and d). A slightly increased NO level was also present after 12 h in A549 cells incubated with 63 T at a concentration of 0.5 μM. Interestingly a clear correlation between the concentration of NO and the 63 T dose was observed in A549 cells for all concentrations. The same trend was observed in CCD39Lu cells only for mild and moderate doses (Fig. 1S).

### Evaluation of MnSOD, CAT, glutathione-S-transferase, glutathione peroxidase activity

Next, we analyzed the activity of GSH-dependent enzymes (GPx, GST), CAT and MnSOD in order to obtain a comprehensive view of the role of antioxidant machinery in cancer and non-cancerous cell lines.

**Fig. 6:**
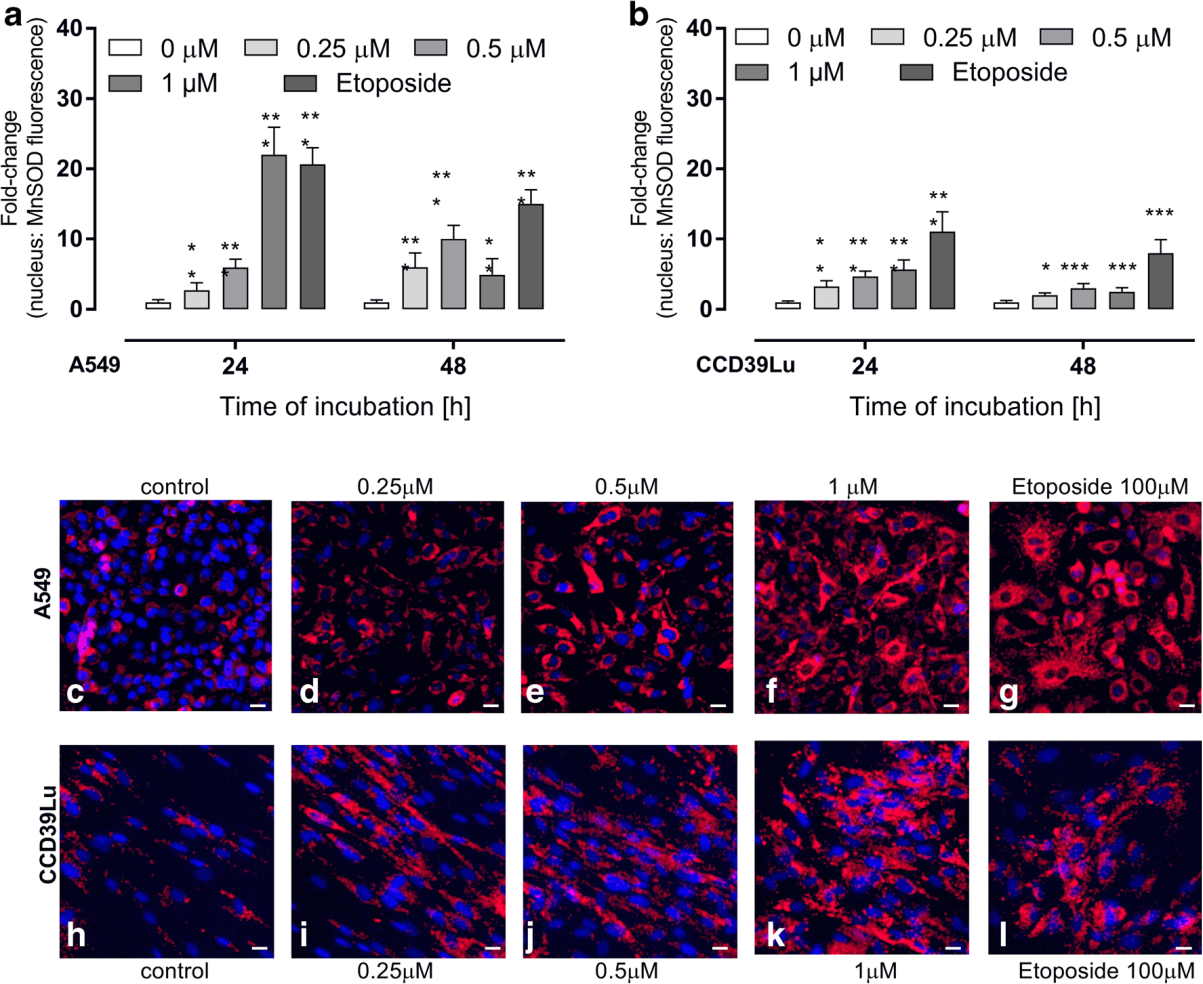
**Activity of manganese dismutase** (**MnSOD) in A549 and CCD39Lu cells after treatment with 63 T.** The fluorescence intensity corresponding to MnSOD activity in A549 (**a**) and CCD39Lu (**b**) after 24 h and 48 h. The representative images were taken after 24 h of incubation with 63 T and present: control A549 cells (**c**) and control CCD39Lu (**d**). The next pictures show A549 cells incubated with 63 T at concentrations of 0.25 μM (**d**), 0.5 μM (**e**), 1 μM (**f**), while pictures **i**, **j**, **k** show CCD39Lu cells incubated with 63 T at concentrations of 0.25 μM, 0.5 μM**,** 1 μM, respectively. Etoposide as a positive control was used at a concentration of 100 μM – pictures (**g**) and (**l**) for A549 and CCD39Lu cells, respectively. Scale bars correspond to 20 μm. Statistical significance is indicated with asterisks: *p < 0.05, **p < 0.01, ***p < 0.001

**Fig. 7:**
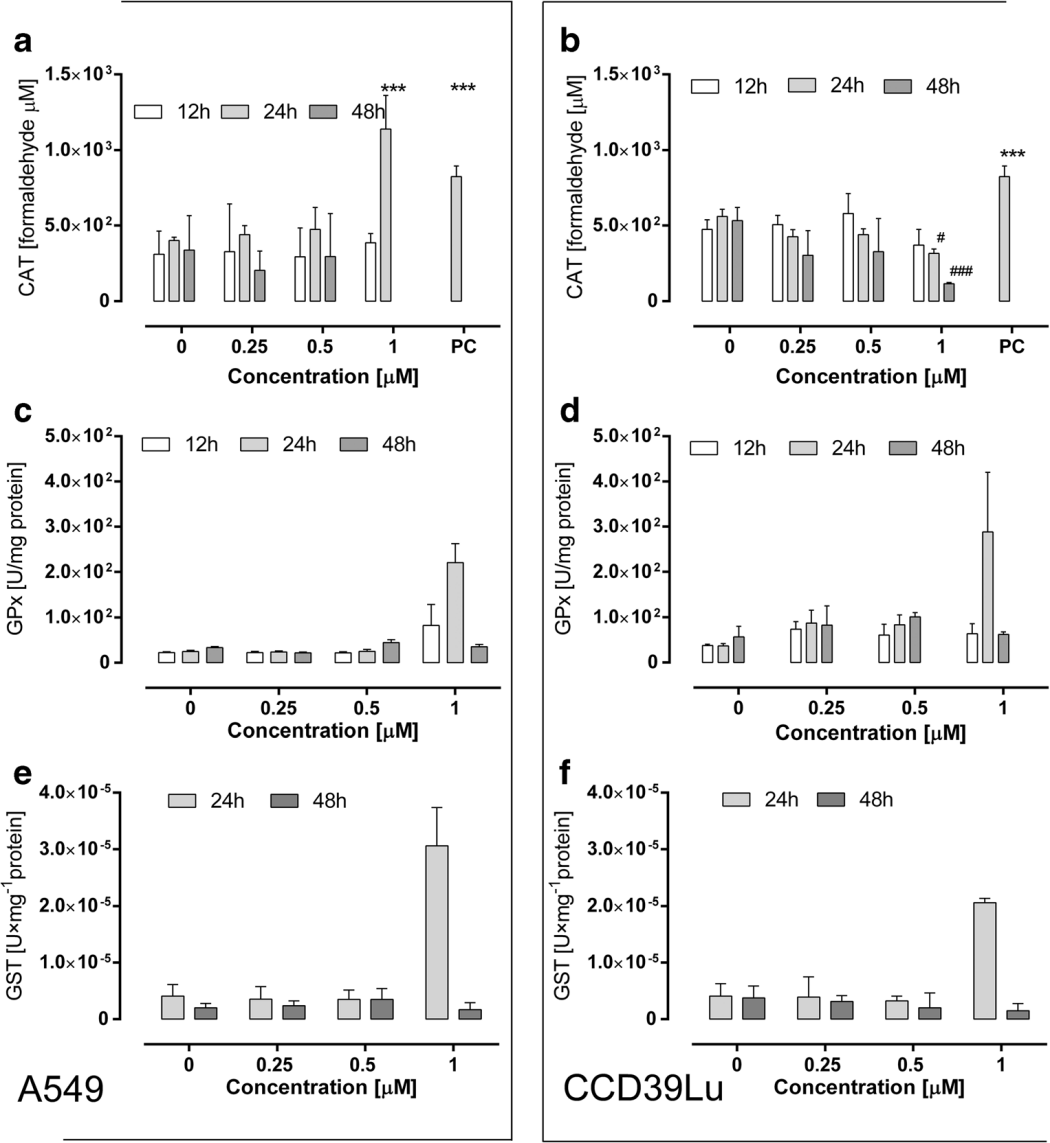
**Impact of incubation with 63 T on CAT, GPx and GST activity.** Panels (**a**) and (**b**) show changes in CAT activity in A549 and CCD39Lu cells, respectively. Panel (**c**) shows the impact of 63 T on GPx activity in A549 cells while panel (**d**) shows GPx activity in CCD39Lu cells incubated with 63 T. Panels (**e**) and (**f**) show the effect of incubation with 63 T on GST activity in A549 and CCD39Lu cell lines, respectively. Cells were treated with 63 T at concentrations of 0.25 μM, 0.5 μM and 1 μM for 12 h, 24 h and 48 h. Statistical significance corresponding to increased activity is indicated with asterisks: *p < 0.05, **p < 0.01, ***p < 0.001, while statistical significance of a decrease in activity is shown with hashtags #*p* < 0.05, ###*p*< 0.001

MnSOD activity was analyzed using a MnSOD Assay. A sample of images is presented in Fig. 6. Data are also presented as histograms (analyzed using Nikon BR software). The blue (nuclei) to red (MnSOD) ratios were calculated and presented in charts. Using this method, a dose-dependent increase in MnSOD activity in A549 cells incubated with 63 T was noticed, while, in adjacent CCD39Lu cells, a significant increase of MnSOD activity was found in cells incubated with 63 T at the higher concentration (1 μM) **(**Fig. 6**)**. The CAT activity was significantly increased only in A549 cells incubated with 63 T at a concentration of 1 μM after 24 h, while incubation for 48 h resulted in decreased activity. A significant loss of activity was also observed for CCD39Lu cells incubated with 63 T at a concentration of 1 μM (Fig. 7). The same trend was found for GST and GPx which significantly increased in A549 and CCD39Lu cell lines after incubation with 1 μM of 63 T for 24 h. A significant depletion of enzymatic activity was observed after the next 24 h (Fig. 7). Increased activity of GPx and CAT supports our findings obtained for H_2_O_2_ detection. Indeed, GPx and CAT are known as the two main enzymes involved in H_2_O_2_ detoxification. Therefore, these correlations between experiments suggest that 63 T at a concentration of 1 μM, particularly in cancer cells, may increase activity of CAT and GPx in response to overproduction of H_2_O_2_.

### Assessment of reduced glutathione (GSH) content

**Fig. 8:**
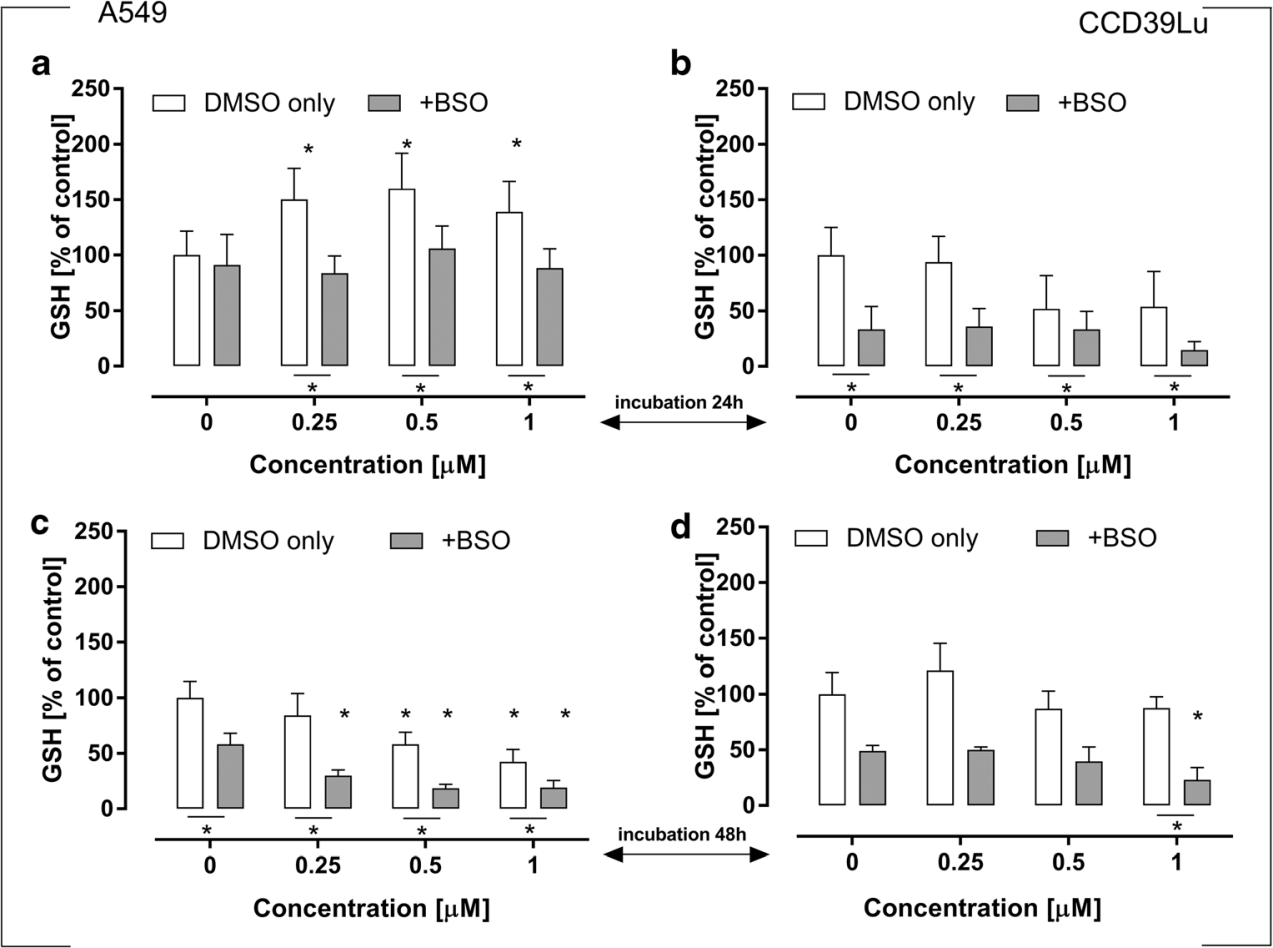
**Changes in intracellular glutathione levels in A549 and CCD39Lu cells** (panels **a, c** and **b, d** respectively) incubated simultaneously with BSO (glutathione synthetase inhibitor) and 63 T alone. Intracellular concentration of GSH in A549 and CCD36Lu was measured after 24 h (panels **a** and **b**) and 48 h (panels **c** and **d**). Statistical significance is indicated with asterisks: *p < 0.05

**Fig. 9:**
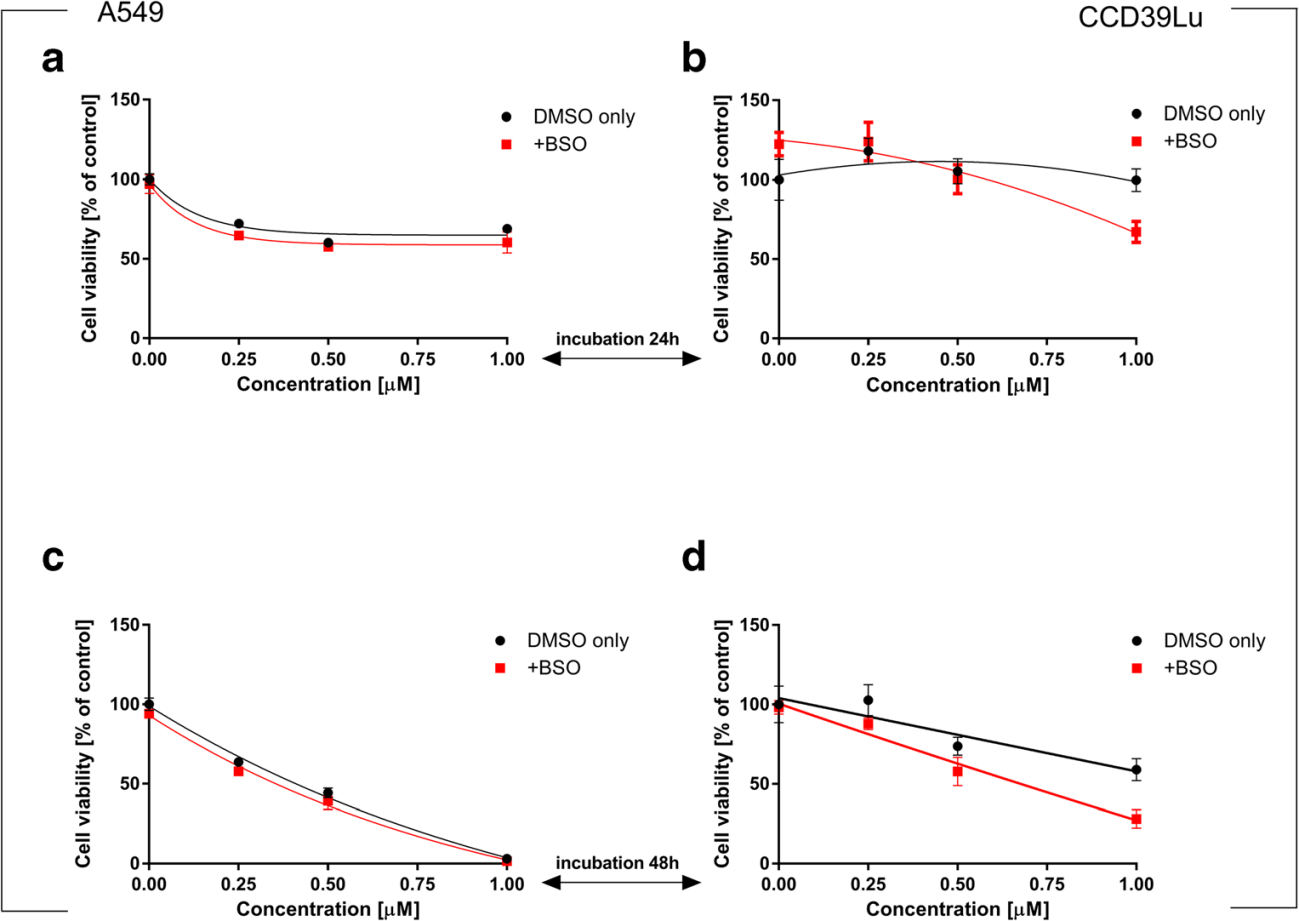
**Effect of GSH synthesis inhibition on A549 and CCD39Lu cells viability.** The impact of BSO co-treatment on cell viability is in A549 cells is shown in panels (**a**) and (**c**), (**a** – after 24 h, **c** – after 48 h), while (**b**) and (**d**) show the parallel effect exerted by 63 T in CCD39Lu cells (**b** – after 24 h, **d** – after 48 h). Statistical significance is indicated with asterisks: *p < 0.05

24 h incubation of A549 cells with 63 T resulted in a significant increase in the GSH level. However, after the next 24 h, a significantly lower level of this important peptide was measured. Interestingly, in CCD39Lu cells the concentration of GSH in cells incubated with 63 T significantly declined after 24 h, while after the next 24 h its level was restored and was similar or even higher in comparison to the concentration measured in the control cells. To confirm the important role of GST in 63 T-mediated oxidative stress induction, BSO was used to inhibit GSH biosynthesis in both cell lines; BSO significantly reduced the concentration of GSH (Figs. 8 and 9).

### The influence of MnSOD, CuZnSOD, 2-ME and glutathione synthetase inhibition on 63 T induced toxicity

**Fig. 10:**
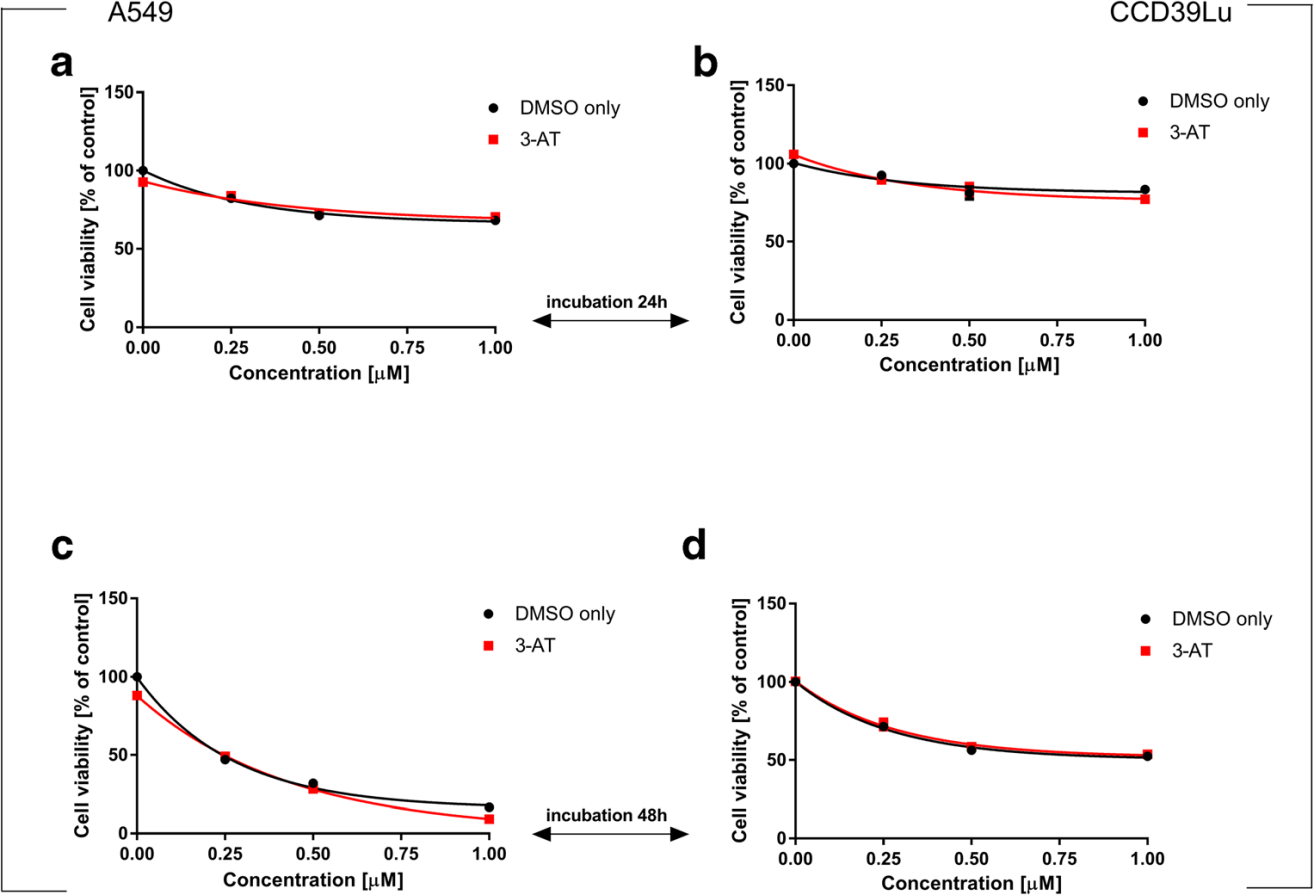
**Synergistic effect of treatment A549 and CCD39Lu with 63 T and CAT inhibitor.** The effect of inhibitor against A549 cells is shown in panels (**a**) and (**c**), (**a** – after 24 h, **c** – after 48 h), while (**b**) and (**d**) show the parallel effect exerted by 63 T in CCD39Lu cells (**b** – after 24 h, **d** – after 48 h). Statistical significance is indicated with asterisks: *p < 0.05

In this study, we used a panel of antioxidative enzymes inhibitors: 3-AT (a **CAT inhibitor)**, DDC (a **CuZn-SOD inhibitor)**, 2-ME (an inhibitor of **Mn-SOD)** and BSO (a **γ-GCS inhibitor),** to investigate whether combining 63 T with a blockage of each of the mentioned enzymes may synergistically potentiate cytotoxicity. We found that 3-AT significantly enhanced the toxicity of 63 T in A549 cells incubated for 48 h at a concentration of 1 μM (Fig. 10). BSO significantly increased the toxicity of 63 T in CCD39Lu lung fibroblasts (Fig. 9) after 48 h of incubation for the dose of 1 μM. This finding showed that the GSH synthesis inhibitor can effectively enhance 63 T activity in CCD39Lu cells and confirmed that the GSH content in non-cancerous cells is important in the anticancer effect of 63 T, as has been suggested by previous studies.

Other tested inhibitors (MnSOD and CuZnSOD inhibitors) did not change the cytotoxic effect of 63 T for both cell lines (Figs. 2S and 3S).

## Discussion

ROS play a crucial role in the death and survival of both normal and cancer cells. ROS production in living cells is controlled by several systems employing antioxidant enzymes such as SOD, CAT or GPx and other small-molecule antioxidants including GSH [32]. The level and cellular turnover of GSH is strictly controlled by several enzymatic systems [33–36]. It is commonly accepted that in cancer cells the enzymatic systems controlling their redox status are even more important than in normal cells, since cancer cells exist in conditions of continuous oxidative stress [37, 38]. The increased ROS generation in cancer cells is linked to a turbulent cellular metabolic activity stimulated by oncogenic signals [39]. Additionally, the oxidative stress in cancer cells may be a consequence of mitochondrial malfunction which is often present in cancer cells [40, 41].

**Fig. 11:**
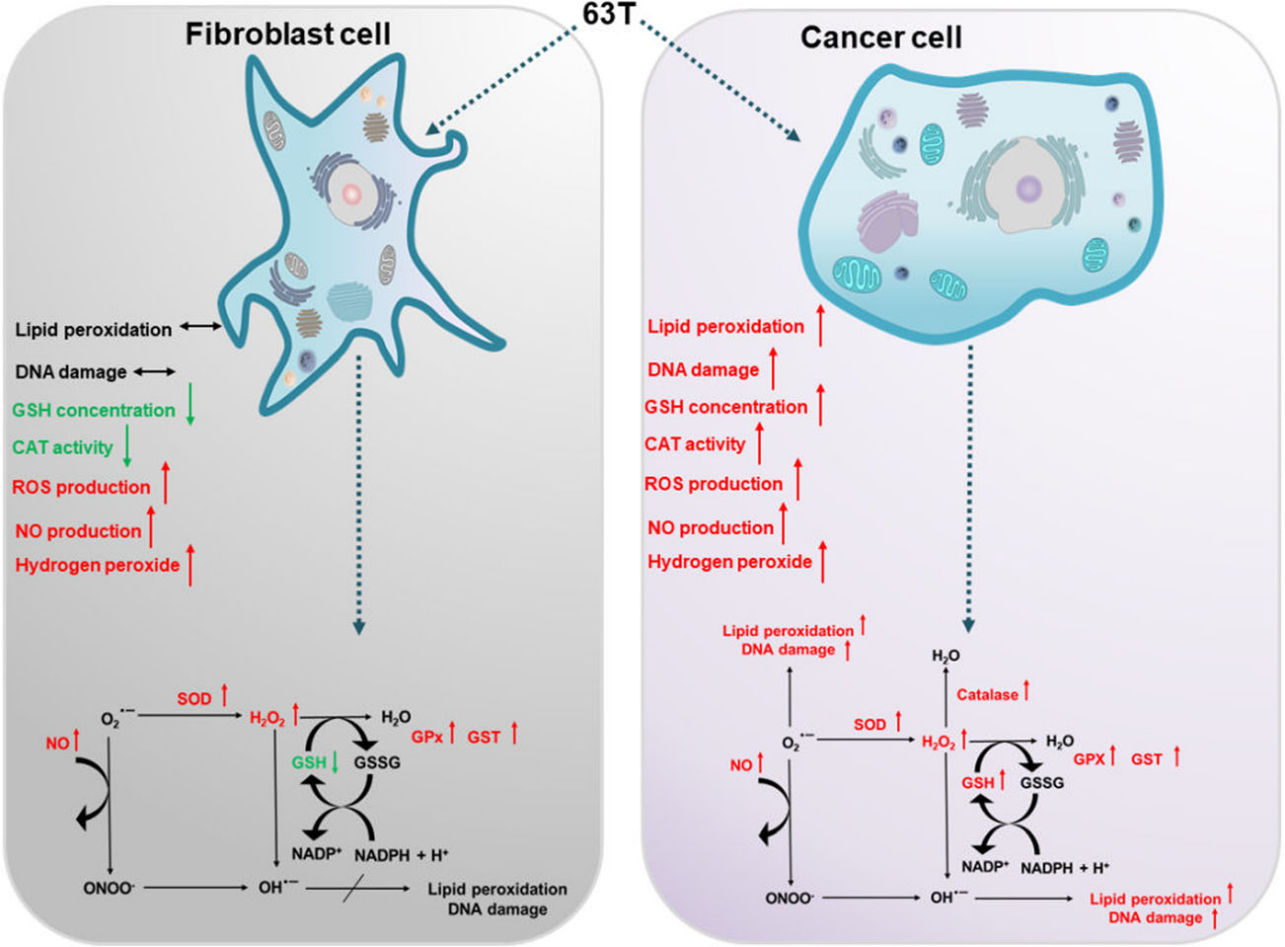
**Response to oxidative stress induced by 63 T in lung cancer and non-cancerous cells.** CAT, catalase; GPx, glutathione peroxidase, GSH, glutathione; GSSG, glutathione disulfide; GST, glutathione-S-transferase; H_2_O_2_, hydrogen peroxide; MnSOD, manganese superoxide dismutase, NO, nitric oxide; O2^.-^, superoxide; OH^.^, hydroxyl radical; ONOO-, peroxynitrite; ROS, reactive oxygen species

The downregulation or inhibition of cellular antioxidative systems with parallel production of ROS may disturb the redox balance in cancer cells and cause apoptotic and/or necrotic death [41–43], which has been previously reported by our group [29, 44]. On the other hand, the overexpression of antioxidative enzymes is observed in some malignant cells as a result of ROS generating anticancer therapy [45]. Our previous experiments performed with A549 and CCD39Lu cells suggested a ROS-driven mechanism of cell death induced by 63 T. Therefore, in this presented study, the impact of this promising benzanilide derivative on the redox balance in A549 lung cancer and CCD39Lu non-tumorigenic lung fibroblasts was investigated. The proposed mechanism and the consequences of oxidative stress in normal fibroblasts and cancer cells line are presented in Fig. 11.

The production of ROS was investigated in a DCHF assay where, in both cell types, an increased fluorescence of DCF was registered using flow cytometry and fluorescence microscopy. In both cell lines, the dose-response analysis suggested different mechanisms of ROS generation and antioxidant defense activity in the two tested types of cells. Since DCF assays conducted using only plate readers or flow cytometry are sometimes criticized due to a lack of morphological analysis [46], additional experiments employing fluorescence microscopy were performed. These experiments discovered “hotspots” in cells and suggest the existence of subcellular structures where ROS generation was particularly intensive (Fig. 3). DCFH-DA is very useful a redox indicator probe that responds to changes in intracellular iron signaling or peroxynitrite formation [47]. Nevertheless, it should be taken into consideration that the intracellular DCF level depends on several factors such as the different esterase activity, a basal redox state as well an expression of MDR (multidrug resistance)-associated ABC transporters [48]. Moreover, the DCF fluorescence intensity may change due to (i) relocation of mitochondrial cytochrome c to cytosol from mitochondrial membrane permeation, (ii) relocation of lysosomal iron to cytosol from lysosomal membrane permeation, (iii) the presence of oxygen, H_2_O_2_ and redox active metals, and (iv) a reaction with oxygen that led to the production of superoxide and, in effect, increased the H_2_O_2_ concentration [46, 48, 49]. Therefore, due to non-specific properties of DA-DCFH, it was used in our study to measure the overall intracellular redox status, and further studies employing more specific redox probes are necessary to fully explain the “hotspots” phenomenon observed by us.

A particularly important finding is that 63 T may selectively induce lipid peroxidation in cancer cells. We observed that the malondialdehyde (MDA) level in cancer cells is relatively higher than in normal fibroblasts. Therefore, 63 T may selectively deregulate redox homeostasis in cancer cells by increasing levels of lipid peroxidation. In general, lipid peroxides act as cytotoxic agents via different mechanisms such as affecting the function of lipid membranes, generating further ROS, modifying DNA and proteins [31]. Our results demonstrated that 63 T may increase MDA production accompanied by the phosphorylation of γH2AX which is a marker of DNA double-strand breaks (DSBs) [50]. The oxidative lesions can lead to DNA DSB formation by different mechanisms: (i) when two DNA single strand breaks (SSBs) form close to each other on opposite strands, (ii) as a result of cleavage sites close to SSBs on the opposite strand by topoisomerases, (iii) when ROS-induced DNA damage affects DNA replication or transcription, and (iv) during the repair of two lesions in a cluster at the same time or when the modified base located close to an unrepaired SSB on the opposite strand is removed [50]. Although the appearance of DSBs is still considered to be less than that of other ROS-induced DNA lesions such as 8-hydroxydeoxyguanosine (8-OHdG), ROS such as H_2_O_2_ may induce H2AX formation [51]. Work by Zhao et al. showed that there is a close association between DNA replication and H2AX phosphorylation in A549 cells [52]. This finding suggested that H2AX phosphorylation may be caused by stalled replication forks and perhaps also by induction of DNA double-strand breaks at the primary DNA lesions induced by H_2_O_2_ [52]. Also, authors reported that activation of Ataxia Telangiectasia Mutated protein kinase (ATM) and induction of H2AX are among the early events of the DDR induced by exposure of cells to H_2_O_2_ in human pulmonary carcinoma A549 cells [52]. On the other hand, Gao et al. found that activation of ATM by H_2_O_2_ in human primary fibroblasts was observed to occur with no evidence of H2AX phosphorylation [53, 54].

Since in our previous studies a significantly high expression of catalase has been measured in cells incubated with 63 T, we have measured hydrogen peroxide production in cells exposed to 63 T. The increased CAT activity may be considered as a possible cellular response to H_2_O_2_ production reported by other authors [55, 56]. In both cell lines incubated with 63 T at a concentration of 1 μM, a significantly increased level of H_2_O_2_ was observed (Fig. 5). It was reported by some authors that the production of H_2_O_2_ in cancer cells incubated with anticancer compounds, e.g. 2-methoxy-6-acetyl-7-methyl juglone, may by followed by increased NO production mainly via the activation of Jun-N-terminal kinase (JNK) and the expression of inducible nitric oxide synthase (iNOS) [48], thereby leading to NO generation in multiple cancer cells or by xanthine oxidoreductase [57, 58]. Therefore, the next molecule to be measured in our experiments was NO. NO is a pleiotropic regulator, synthesized by a few nitric oxide synthetases (NOS - inducible iNOS, endothelial eNOS and neuronal nNOS) and plays a key role in numerous biological processes, including neurotransmission [59], vasodilatation [60], and macrophage-mediated immunity [61]. NOS activity has been detected in tumor cells of various histological origins and has been associated with tumor grade, proliferation rate and expression of important signaling components associated with cancer development such as the estrogen receptor. Consequently, NO has been described as a Janus-faced-molecule [62] which modulates several cancer-related events including angiogenesis, apoptosis, cell cycle, invasion, and metastasis in both cell death and cell survival directions [63]. In our experiment, the level of NO increased in both cell lines in a concentration-dependent manner. Interestingly, the basic concentration of NO was four times higher in CCD39Lu cells (Fig. 5). In general, the lung interstitium consists mainly of fibroblasts which are responsible for the production of extracellular matrix (ECM) proteins and also play an important role during injury repair [64]. A number of other studies show the role of NO produced by fibroblasts during inflammation and wound-repair [65–67]. It was reported that exposure to a different proinflammatory stimulus (LPS, IFN, cytokine mix) may modify the response of lung epithelial cells to produce nitric oxide. Importantly, when lung fibroblasts were co-cultured with cancer cells, it was observed that the NO production was associated with a higher ratio of fibroblasts [66]. Moreover, as mentioned by White et al., NO and NO donors increase cellular GSH in a rat lung fibroblast cell line (RFL6 cells) and, thus, NO could have a protective effect against oxidant injury by increasing cellular GSH synthesis [68].

In the case of ROS overproduction and induction of oxidative stress, the compensative mechanism of antioxidative defense is expected [45]. The basic enzymatic cellular antioxidative system comprises several strictly collaborating enzymes: superoxide dismutase, CAT, and GPx. The mitochondria are the most sensitive cellular organelles where free radicals may induce cell death. In our experiments, a significant increase in MnSOD activity was observed in A549 cells, while in CCD39Lu cells its increase was less efficient. Although mitochondria are equipped with a relatively efficient antioxidant system, they are generally devoid of CAT. However, the expression of this enzyme, which is very important in the regulation of redox status, is very high in the cytoplasm. A significant induction of CAT was shown in our previous studies using immunoassay [25]; however, in the present study an assay measuring CAT enzymatic activity in cell lysates was used. Using this assay, a very high induction of CAT was noted only in A549 cells incubated for 24 h with 63 T at a concentration of 1 μM. Similarly, high activity of two key glutathione-dependent enzymes, GPx and GST, was measured in cells incubated for 24 h with 63 T. Analogously to CAT, the activity of these enzymes declined sharply after the next 24 h suggesting progression in the disintegration of cellular structures and the degradation of enzymes followed by cell death.

Since a significant increase of GSH-dependent redox buffers was observed as a result of 63 T toxicity, we further assayed the effect of this compound on the GSH level. In order to evaluate GSH recycling machinery, the GSH synthesis inhibitor BSO was used. As shown in Fig. 8**,** the reaction of GSH synthesis in A549 cells was very fast and could be observed after 24 h. The synthesis of new GSH was efficiently inhibited by BSO. In CCD39Lu cells, a significant decrease in the level of GSH was followed by its resynthesis which was observed after 48 h of incubation. It was also observed that CCD39Lu cells were much more sensitive to BSO treatment and, in these cells, BSO significantly potentiated the cytotoxic activity of 63 T, while in A549 cells the synergistic action of 63 T and BSO was not observed. This result suggests significant differences in ROS defense systems in both cell lines.

Following this link, we assayed the impact of a few inhibitors of antioxidant enzymes: 2-ME for MnSOD, DDC for CuZnSOD and 3-AT for CAT on the cytotoxicity of 63 T. From these inhibitors, only 3-AT slightly potentiated the cytotoxic activity of 63 T, again indicating the role of H_2_O_2_ in the cytotoxic activity of 63 T.

In conclusion, it may be summarized from this stage of experiments that 63 T is absorbed by cancer cells and non-tumorigenic cells and is probably metabolized to short living reactive metabolites. The incubation of tested cells with 63 T resulted in changes in the expression of several antioxidative and drug-metabolizing enzymes as well as enzymes responsible for cell death or survival. It should be stressed that the concentration range used in our experiments was very narrow, however, the observed cytotoxic effects ranged from relatively mild effects at 0.25 μM through significant effects observed at 0.5 μM to severe toxicity observed in cells incubated with 63 T at a concentration of 1 μM, where the whole cellular machinery was quickly inactivated and destroyed leading to cell death. The selective activity against cancer cells showed in our experiments is caused at least partially by the different response for ROS in both tested cell lines as well as different metabolic pathways leading to oxidative stress. In further studies, metabolic pathways involved in the formation of reactive metabolites as well the pathways involved in their detoxification should be investigated and explained. Additionally, metabolites responsible for the cytotoxic activity of 63 T should be identified.

## Electronic supplementary material

Figure 1S 63 T induces dose-dependent generation of NO. The graph showing a linear relationship between a concentration of 63 T and NO generation, r-squared linear-coefficient of determination. (PNG 156 kb)

High resolution image (TIF 1801 kb)

Figure 2S The effect of MnSOD inhibitor on 63 T activity. The effect of an inhibitor on A549 cells is shown in panels (A) and (C), (A – after 24 h, C – after 48 h), while (B) and (D) show the parallel effect exerted by 63 T in CCD39Lu cells (B – after 24 h, D – after 48 h). (PNG 340 kb)

High resolution image (TIF 863 kb)

Figure 3S The cytotoxic activity of 63 T is not affected by the Zn/CuSOD inhibitor in A549 (A – after 24 h, C – after 48 h) and CCD39Lu (B – after 24 h, D – after 48 h) cell lines. (PNG 360 kb)

High resolution image (TIF 945 kb)

## Abbreviations

2ME: 2-methoxyestradiol
3AT: 3-Amino-1,2,4-triazole
BCA: Bicinchoninic acid assay
BHT: Butylated hydroxytoluene
BSO: Buthionine sulphoximine
CAT: Catalase
CDNB: 1-chloro-2,4-dinitrobenzene
DAF-FM DA: 4-Amino-5-methylamino- 2′,7′-difluorofluorescein diacetate
DCF: 2,7-dichlorofluorescein
DDC: Diethyldithiocarbamate
DMEM: Dulbecco’s modified Eagle’s medium
DMSO: Dimethyl sulfoxide
eNOS: Endothelial nitric oxide synthetase
FACS: Fluorescence-activated cell sorting
FBS: Fetal bovine serum
GPx: Glutathione peroxidase
GSH: Glutathione
H_2_DCF: Dihydrodichlorofluorescein
H2DCF-DA: Dihydrodichlorofluorescein diacetate
iNOS: Inducible nitric oxide synthetase
JNK: Jun-N-terminal kinase
MDA: Malondialdehyde
MTT: 3-(4,5-dimethylthiazol-2-yl)-2,5-diphenyltetrazolium bromide
NADPH: β-Nicotinamide adenine dinucleotide 2′-phosphate reduced tetrasodium salt hydrate
nNOS: Neuronal nitric oxide synthetase
NOS: Nitric oxide synthetase
PBS: Phosphate buffered saline
PUFAs: Polyunsaturated lipids fatty acids
RNS: Reactive nitrogen species
ROS: Reactive oxygen species
SD: Standard deviation
SOD: Superoxide dismutase
TBA: Thiobarbituric acid
