# Supplementary figures and images for: The role of oxidative stress in 63 T-induced cytotoxicity against human lung cancer and normal lung fibroblast cell lines
Source: Invest New Drugs. 2018 Nov 29;37(5):849–64. doi: 10.1007/s10637-018-0704-8 (PMC6736908; doi:10.1007/s10637-018-0704-8)

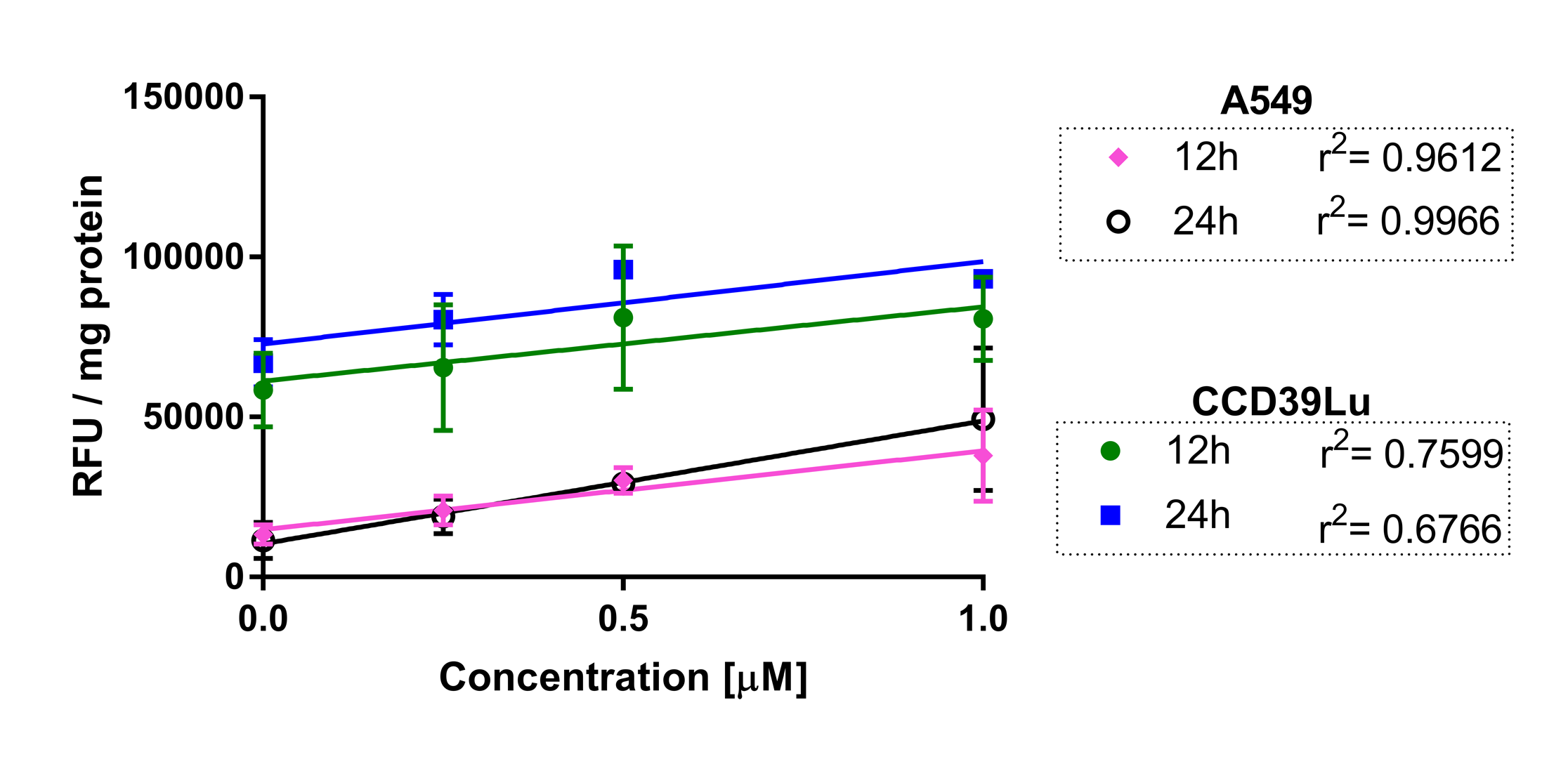

Supplement: Supplementary file 1 — 63 T induces dose-dependent generation of NO. The graph showing a linear relationship between a concentration of 63 T and NO generation, r-squared linear-coefficient of determination. (PNG 156 kb) [file 10637_2018_704_Fig12_ESM.png]

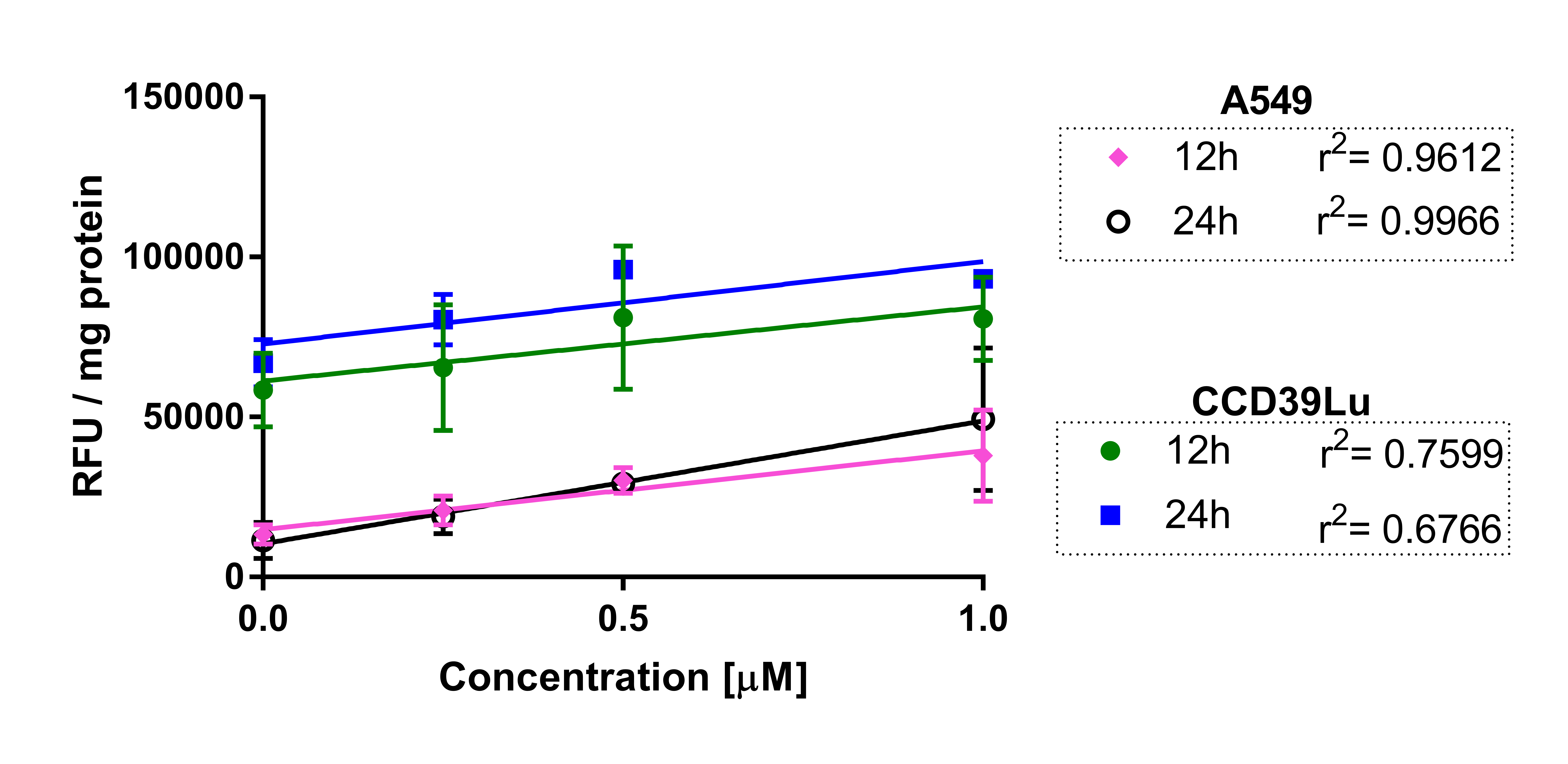

Supplement: Supplementary file 2 — High resolution image (TIF 1801 kb) [file 10637_2018_704_MOESM1_ESM.tif]

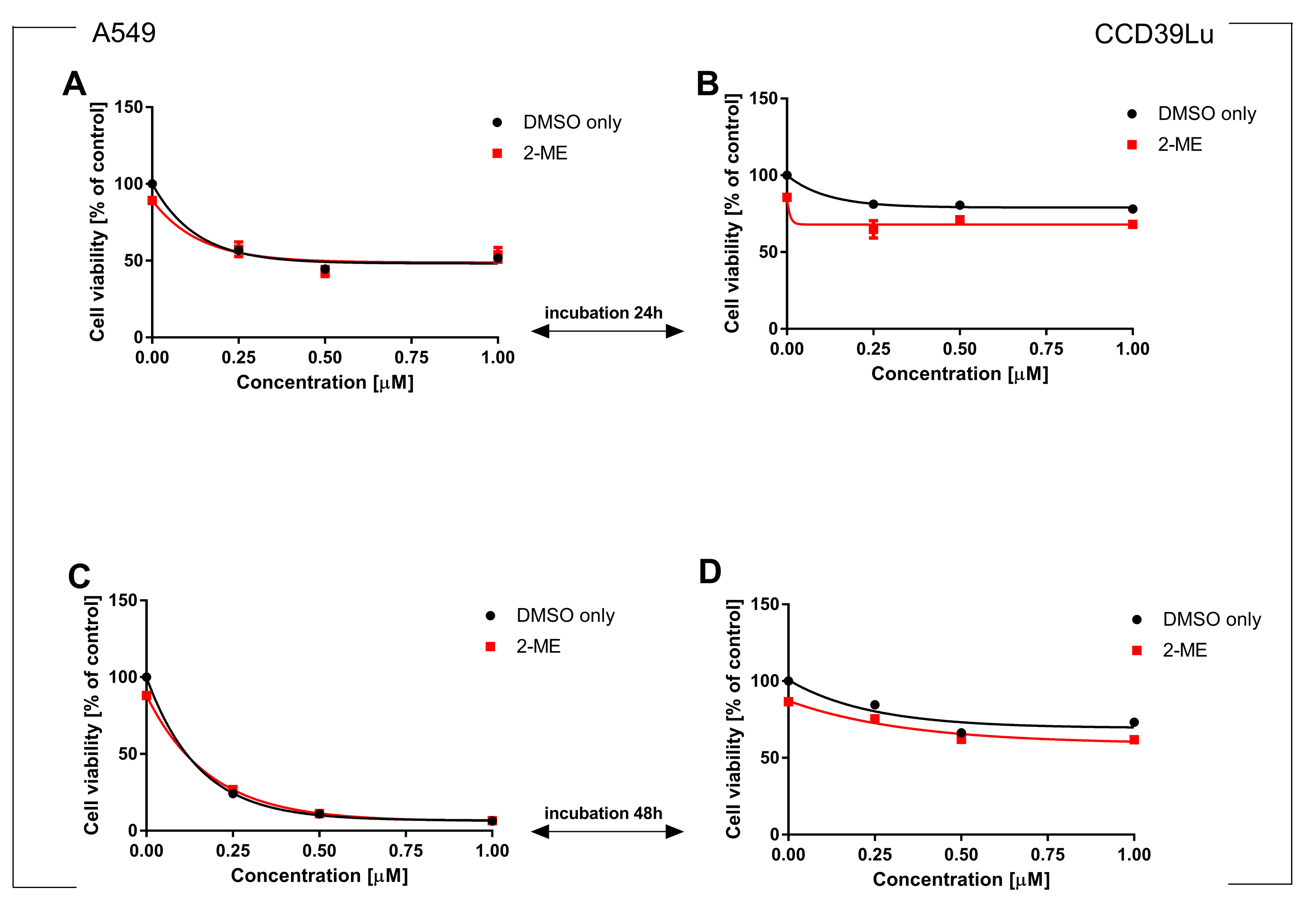

Supplement: Supplementary file 3 — The effect of MnSOD inhibitor on 63 T activity. The effect of an inhibitor on A549 cells is shown in panels (A) and (C), (A – after 24 h, C – after 48 h), while (B) and (D) show the parallel effect exerted by 63 T in CCD39Lu cells (B – after 24 h, D – after 48 h). (PNG 340 kb) [file 10637_2018_704_Fig13_ESM.png]

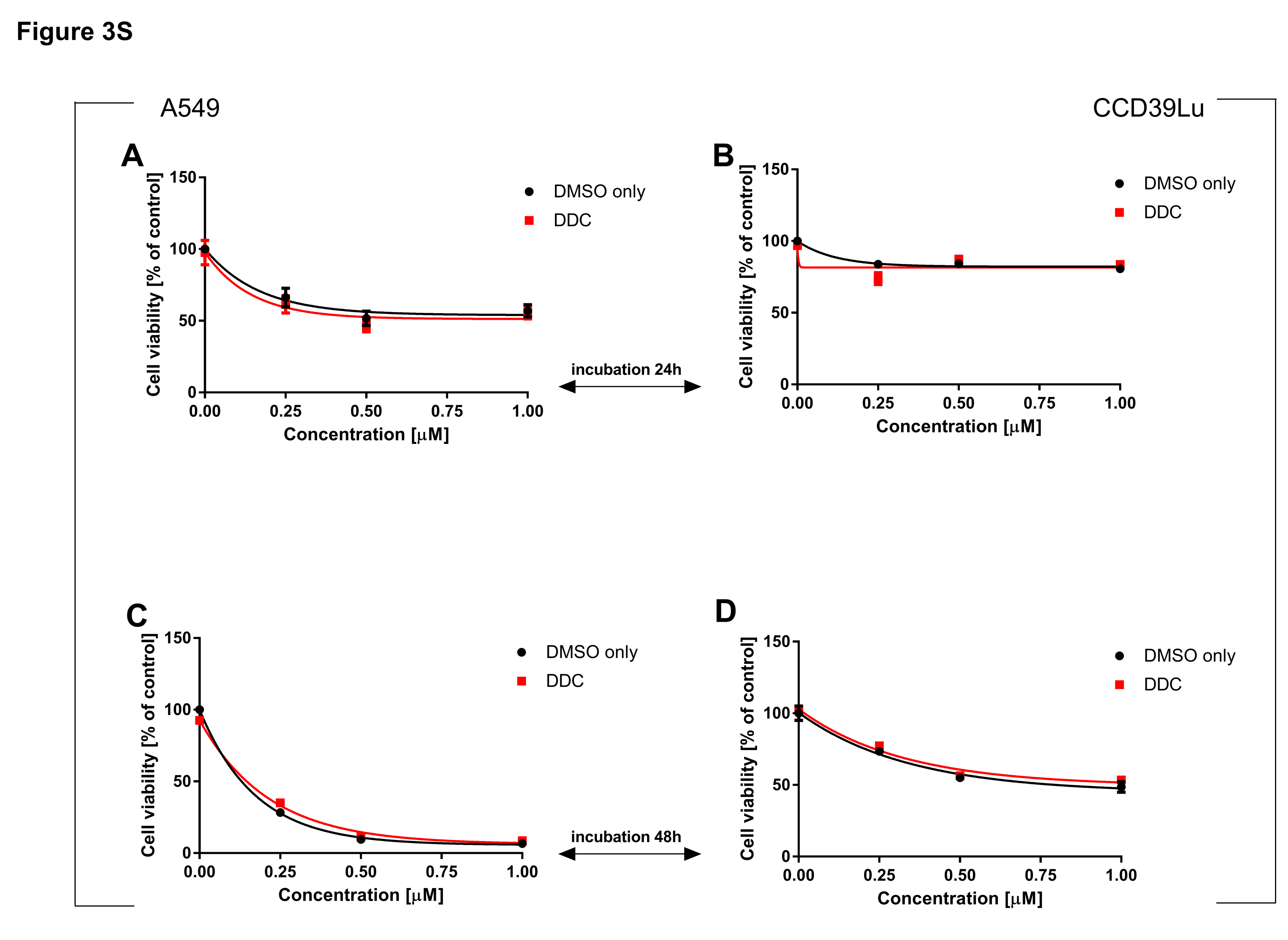

Supplement: Supplementary file 5 — The cytotoxic activity of 63 T is not affected by the Zn/CuSOD inhibitor in A549 (A – after 24 h, C – after 48 h) and CCD39Lu (B – after 24 h, D – after 48 h) cell lines. (PNG 360 kb) [file 10637_2018_704_Fig14_ESM.png]
